# Are German patients burdened by the practice charge for physician visits ('Praxisgebuehr')? A cross sectional analysis of socio-economic and health related factors

**DOI:** 10.1186/1472-6963-8-232

**Published:** 2008-11-12

**Authors:** Ina-Maria Rückert, Jan Böcken, Andreas Mielck

**Affiliations:** 1 Helmholtz Zentrum Muenchen – German Research Center for Environmental Health, Institute of Epidemiology, Neuherberg, Ingolstädter Landstrasse 1, 85764 Neuherberg, Germany; 2 Bertelsmann Stiftung, Carl-Bertelsmann-Straße 256, 33311 Gütersloh, Germany; 3 Helmholtz Zentrum Muenchen – German Research Center for Environmental Health, Institute of Health Economics and Health Care Management, Neuherberg, Ingolstädter Landstrasse 1, 85764 Neuherberg, Germany

## Abstract

**Background:**

In 2004, a practice charge for physician visits ('Praxisgebuehr') was implemented in the German health care system, mainly in order to reduce expenditures of sickness funds by reducing outpatient physician visits. In the statutory sickness funds, all adults now have to pay € 10 at their first physician visit in each 3 month period, except for vaccinations and preventive services. This study looks at the effect of this new patient fee on delaying or avoiding physician visits, with a special emphasis on different income groups.

**Methods:**

Six representative surveys (conducted between 2004 and 2006) of the Bertelsmann Healthcare Monitor were analysed, comprising 7,769 women and men aged 18 to 79 years. The analyses are based on stratified analyses and logistic regression models, including a focus on the subgroup having a chronic disease.

**Results:**

Two results can be highlighted. First, avoiding or delaying a physician visit due to this fee is seen most often among younger and healthier adults. Second, those in the lowest income group are much more affected in this way than the better of. The multivariate analysis in the subgroup of respondents having a chronic disease shows, for example, that this reaction is reported 2.45 times more often in the lowest income group than in the highest income group (95% CI: 1.90–3.15).

**Conclusion:**

The analyses indicate that the effects of the practice charge differ by socio-economic group. It would be important to assess these effects in more detail, especially the effects on health care quality and health outcomes. It can be assumed, however, that avoiding or delaying physician visits jeopardizes both, and that health inequalities are increasing due to the practice charge.

## Background

The German health care system has experienced several reforms in the past years, usually shifting more financial responsibility to the insured, e.g. by raising co-payments for prescribed drugs [1]. The analyses presented here focus on a relatively new reform. Starting on January 1^st ^2004, a practice charge ('Praxisgebuehr') of € 10 for the first contact at a physician's or dentist's office in each 3 month period was introduced as part of the 'Statutory Health Insurance Modernization Law' (§28, clause 4, SGB V). All statutory insured, aged 18 years or older, are affected by this new arrangement. Approximately 90% of the German population has statutory health insurance while the remaining 10% (mainly self employed, civil servants and high income groups) are covered by private health insurance.

The practice fee is not charged for preventive medical services, such as cancer screenings, examinations to ensure a normal pregnancy, general health checks (for people above 35 years of age) and dental prophylaxis. It is paid in cash at the doctor's office, then sent to the statutory health insurance (i.e. the physician has to ask the patient for this money, but cannot keep it). In the statutory health insurance, there is an upper limit for the annual out-of-pocket payments, i.e. no insured has to pay more than 2% of his or her gross annual income. For those with a chronic disease, the upper limit has been reduced to 1%. The practice charge is added to other out-of-pocket payments (e.g. for drugs or for dentures), and it is assumed that it does not put undue financial burden on the insured. It is important to point out that there is inadequate empirical data supporting this assumption. H. Reiners and M. Schnee, though, analyzed data of the Healthcare Monitor in 2007 and their results suggest that the effects of the practice fee are not distributed evenly [2]. Apparently, people with a lower socio-economic status are more affected and clearly tend to avoid or delay physician visits because of the fee.

The introduction of a fee for physician visits has been justified by three arguments: first, utilization of ambulatory medical services is rather high in Germany [3]. The fee is intended to discourage patients from inappropriate (i.e. superfluous) physician visits. The theorem of 'moral hazard' states that the insured in a statutory health insurance have no incentives for reducing their health care utilization, but try to make the most of their given financial contribution. It has often been quoted for justifying the necessity of co-payments, and the same has been true for the practice charge. Second, the fee helps to reinforce the 'gate keeper' role of the general practitioner (GP), as the GP can decide whether the patient should see a specialist, and as the patient has to pay the fee only once in each 3 months period (i.e. if referred, no additional fee has to be paid). Third, it is expected that the expenditures of the statutory health insurance will be reduced, at least in the short run. This financial gain is thoroughly wished for, as limiting the expenditures has been a major objective of all past health care reforms in Germany.

Today, more than four years after its implementation, discussion about the effects of the practice charge is still highly controversial. The major questions are, whether 'important' physician visits are reduced (not just 'unimportant' ones) and whether socio-economic inequalities are intensified, as it is well known in Germany that morbidity and mortality are greater in low status groups [4], and that these groups are more heavily affected by additional financial burdens. There are very few studies looking at potential effects in more detail, they are using different data sources and they reach different conclusions. A survey of the Bertelsmann foundation (Healthcare Monitor) in spring 2004 (i.e. shortly after the introduction of the practice charge) shows: 55% of the insured say that the new fee has influenced their health care behaviour in some way, and among these 27% report delayed physician visits, while 35% avoided them completely and tried to cure themselves. Also, 38% had an additional visit in order to obtain a referral to a specialist. Until autumn 2005, the proportion of 'delayers' increased to 42%, while the proportion of 'avoiders' declined to 25%. Also, the average number of physician visits decreased by 8% from 2003 to 2005 [5-7].

Another study conducted by the Scientific Institute (WIdO) of the largest statutory sickness fund (i.e. the AOK) found that in 2004 11.7% of the insured delayed a physician visit or avoided it altogether because of the additional fee [8]. In the following year, this proportion declined to 9.4% [9]. Also, the number of referrals increased between 2004 and 2005. Finally, an analysis of data from the 'Socioeconomic Panel (SOEP)' for the period between 2003 and 2005 did not find any significant effects of the fee on patient behaviour, which may be due to the fact that a very different source of data was used [10].

It is important to stress that the question of whether the fee puts a special burden on the insured from low status groups has yet to be fully answered. There are some hints indicating that low status groups are more influenced than high status groups, for example the Healthcare Monitor surveys and the study 'Living in Europe 2005' of the German Federal Statistical Office [11]. The analyses based on the SOEP study [10] and the WIdO study in 2005 [9] did not find any differences between socio-economic groups, but these analyses have been rather simple and purely descriptive. Some papers have been published on the potential effects of consultation fees and co-payments [12], however there is a special need for more extensive empirical analyses. The study presented here aims at providing more detailed information on the influence of the consultation fee on patient behaviour, with a special focus on differences by socio-economic status. Our study hypothesis is that patients who are socio-economically disadvantaged will delay or even avoid physician visits to a larger extent than patients with more favourable socio-economic backgrounds.

## Methods

The Bertelsmann foundation is an independent, non profit foundation, focussing on its own projects according to the objective of its founder [13]. Since 2001, it has conducted the 'Bertelsmann Healthcare Monitor', a series of surveys in a representative sample of the German population. Two surveys are conducted each year, one in spring and one in autumn. The sampling procedure is embedded in the 'German TNS Infratest Access Panel', comprising a representative sample of about 180,000 adults speaking German aged 18 to 79. From this pool, new independent cross sectional samples are drawn for every survey. The questionnaire for the Healthcare Monitor surveys includes about 26 pages and 130 questions. It is mailed to the participants. A group of basic questions is always identical; the other questions vary according to new research topics. The average response rate is about 70%, resulting in data from approximately 1,500 respondents per survey. The quality of the data is assured by a permanent monitoring process [14]. Thus, the Bertelsmann Healthcare Monitor has been established in the scientific field as a good and important data source, and a number of publications have already been based on it (e.g. [15-18]).

The German Infratest Access Panel has been compared, for example, with data of the Statistical Yearbook of Germany. The comparison shows a very good match with the age structure and with the distribution of the population in the administrative regions (as they are defined by the Federal Agency of Statistics). A good match can also be seen with the gender-specific rates of hospital discharge statistics, and with the prevalence of self reported medical conditions as assessed by the German National Health Survey [19]. The Healthcare Monitor draws cross sectional samples from the Access Panel that are representative for the German population concerning age, gender and region.

The questionnaire has not been supervised by an ethics committee, since the Healthcare Monitor is not a clinical trial; thus, ethical approval was not required. TNS Infratest follows the guidelines of the ADM (official group of the German market research of social science institutes) as well as the European standards of the ESOMAR and the German data protection directive.

All available surveys including the following question on the new patient fee are comprised in the analyses, i.e. surveys 6 (spring 2004) to 11 (autumn 2006). The question reads: Because of the practice charge, and during the past 3 months (i.e. 'quarter'), did you (a) delay a physician contact [e.g. wait for the near end of the quarter], (b) avoid a physician contact [and cure yourself without professional treatment] or (c) make an additional physician contact [in order to obtain a referral to a specialist]? Not marking any of these three categories indicates that the fee had no influence on the participant's behaviour concerning physician visits. The analyses focus on those who did mark one of the three answers, and it should be noted that these three categories are mutually exclusive. Thus, the dependent variable is the percentage of participants who have delayed or avoided a physician visit due the patient fee, among all participants who have answered the questionnaire.

The following variables are included as well:

- per capita net household income per month: five groups, each covering about 20% of the sample (included as the main independent variable)

- number of the survey (six waves from spring 2004 to autumn 2006)

- age (six groups)

- gender

- difficulty to pay co-payments: This question has three answer categories: not difficult, somewhat difficult, very difficult. The corresponding question reads: 'Is it difficult for you to afford co-payments for prescribed drugs?'

- health awareness: The participants were asked how much attention they generally pay to their health, and could tick one of the following categories: very much, much (these two categories are combined in the analysis to 'strong health awareness'), medium, little, no attention at all (the last two categories are combined to 'little health awareness').

- self assessed health: This question has five answer categories that were combined to three categories indicating good, medium or poor health.

- presence of a chronic disease (yes, no): The questionnaire asks about the presence of 18 different chronic diseases (e.g. hypertension, myocardial infarction, asthma). If at least one of them was marked, the respondent was characterized as having a chronic disease.

- reduction of the maximum co-payment to 1% of the income due to a chronic disease (yes, no): As already mentioned above, insured with a chronic disease have to pay only a maximum of 1% of their gross income for co-payments (and not 2% as the other insured), but first the statutory sickness fund has to accept their application for this reduced upper limit.

The analyses are restricted to those participants who were insured in the statutory sickness funds. The association between the dependent and the independent variables are first tested by Chi-square statistics. Only those independent variables yielding a significant association (p = 0.05) are kept for further multivariate analyses. Multicollinearity is tested by the variance inflation factor (VIF), with values above 5.0 indicating problems of multicollinearity [20]. Logistic regression models were computed including or excluding the variable 'difficulty to pay co-payments', and including all insured or only those with a chronic disease. In order to additionally assess the influence of missing values, complete case analyses were conducted and analyses including missing values by separate dummy categories. The quality of the logistic regression models is assessed by the c-value, and the Hosmer Lemeshow Test. The c-value describes the area under the ROC-curve. A value of 1.0 indicates that all cases are classified correctly, while 0.5 marks a model of random correlations. In epidemiologic studies, values between 0.6 and 0.8 are usually regarded as being satisfactory. The Hosmer Lemeshow test is applied to all models. Observed and predicted values are compared with each other, and if there is no significant difference (at the significance level of 0.05) the model is characterised as being appropriate. Finally, Nagelkerke's and McFadden's pseudo-R^2^s have been computed in order to give an impression of the variance explained by the variables in the models, a value of 0 indicating no and of 1 indicating full model fit. The software 'SAS Version 9.13' has been used in all analyses.

## Results

### Univariate models

The data set, including surveys 6 (spring 2004) to 11 (autumn 2006), comprises people covered by different insurance funds and one person aged 17. We included only those who were 18 years of age or older and insured in statutory sickness funds, i.e. 7,769 individuals. 505 respondents (6.50%) did not give the information on their income and another 66 (0.85%) did not answer one of the other questions included in these analyses.

The respondents who did not answer the question on their income were compared to the other respondents who provided this information. The results show that the former somewhat less often avoided or delayed a physician visit due the 'Praxisgebuehr', that they less often had to pay a maximum of just 1% of their income for co-payments, and that they more often said that paying the co-payments was no big problem for them. Furthermore, non-reporting of income was seen more often for women than for men. All other variables show no significant differences between those who gave the information on income and those who did not. It is concluded that missing values for income do not occur completely at random, that it is often the higher income groups and the more healthy participants who do not provide this information. As the number of missing values is rather small, the bias introduced can probably be neglected, though.

The characteristics of the study population are shown in Table 1. Since the distribution of the variables remained rather stable across the different surveys (except the outcome variable), we report only percentages based on the total sample including all six surveys.

**Table 1:** Characteristics of the study population

		**N miss**	**N**	**n**	**%**
*Survey*		0	7,769		
	spring 2004			1,393	17.93
	autumn 2004			1,260	16.22
	spring 2005			1,343	17.29
	autumn 2005			1,273	16.39
	spring 2006			1,218	15.68
	autumn 2006			1,282	16.50
***Socio-economic variables***					
*Age (years)*		0	7,769		
	18–30			1,146	14.75
	31–40			1,501	19.32
	41–50			1,543	19.86
	51–60			1,241	15.97
	61–70			1,561	20.09
	71–79			777	10.00
*Gender*		0	7,769		
	female			4,508	58.03
	male			3,261	41.97
*Per capita income per month (Euro)*		505	7,264		
	< 600			1,659	22.84
	600–800			1,582	21.78
	800–1,000			1,141	15.71
	1,000–1,300			1,363	18.76
	> 1,300			1,519	20.91
*Co-payments*		192	7,577		
	very difficult			1,765	23.29
	somewhat difficult			2,841	37.50
	not difficult			2,971	39.21
***Health related variables***					
*Health awareness (HAW)*		38	7,731		
	strong			3,854	49.85
	medium			3,360	43.46
	little			517	6.69
*Self assessed health (SAH)*		36	7,733		
	good			2,055	26.57
	medium			3,961	51.22
	poor			1,717	22.20
*Chronic disease*		0	7,769		
	yes			3,817	49.13
	no			3,952	50.87
*Maximum co-payment of 1%*^*a*^		0	7,769		
	yes			1,096	14.11
	no			6,673	85.89
***Outcome variable due to practice charge***		0	7,769		
*effect 'yes' {*	delayed physician visit			2,075	26.71
	avoided physician visit			1,400	18.02
	additional physician visit			1,937	24.93
*effect 'no'*	none of the above			2,357	30.34

^*a *^accepted by the sickness fund as being chronically sick

**Figure 1:**
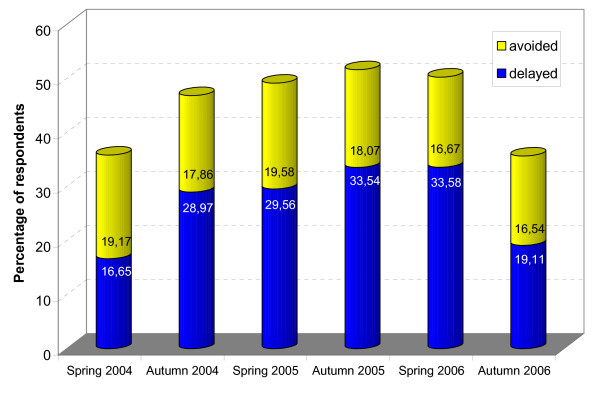
Percentage of participants who have avoided or delayed physician visits due to the practice charge.

Concerning the outcome variable, about 27% of all participants delayed a physician visit, 18% avoided a visit, 25% made an additional visit, and 30% did not report any change in their behaviour due to the practice charge. Over the course of the six surveys, the proportion of delayers and avoiders changed considerably (see Figure 1), notably the percentage of delayers with a peak in autumn 2005 and spring 2006, while the percentage of avoiders remained relatively constant at about 18%.

**Figure 2:**
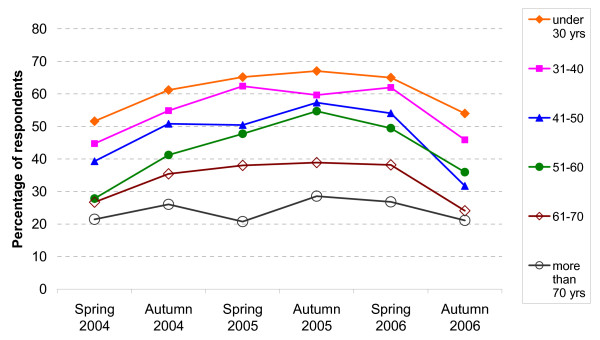
Avoided or delayed physician visits: by age.

**Figure 3:**
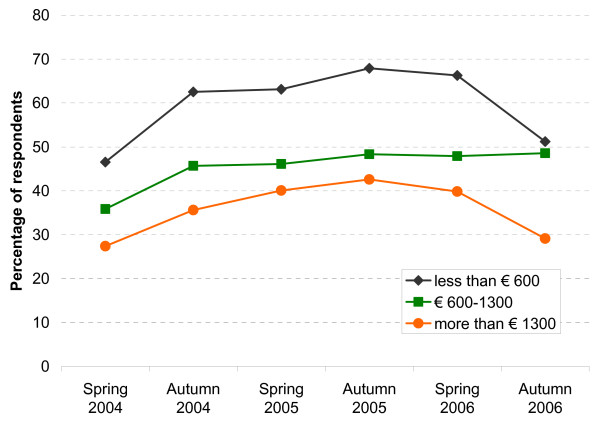
Avoided or delayed physician visits: by per capita income.

**Figure 4:**
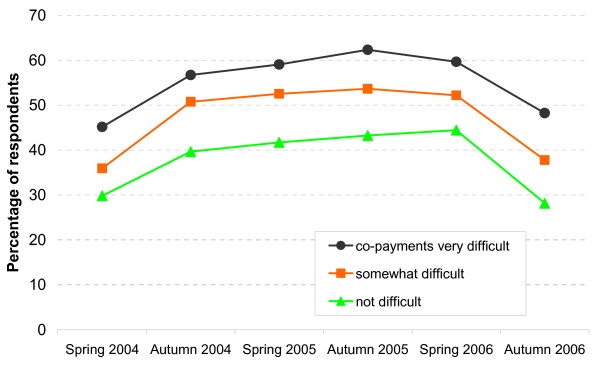
Avoided or delayed physician visits: by difficulty to pay co-payments.

**Figure 5:**
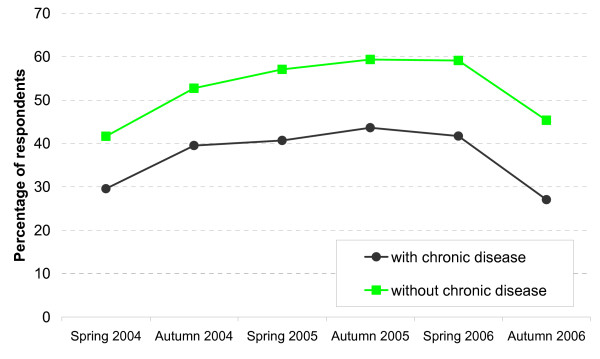
Avoided or delayed physician visits: by presence of chronic disease.

The percentage of respondents reporting delayed or avoided physician contacts is calculated for each survey, for different age groups, income groups, by difficulty to pay the co-payments and for participants with or without a chronic disease (see Figures 2 to 5). As illustrated in Figure 2, avoiding or delaying physician contacts strongly depends on the age of the participants: the percentage clearly decreases with increasing age and the difference between the age groups is statistically significant (chi^2^/5 degrees of freedom = 399.43, p-value < 0.0001; univariate logistic model: under 30 years versus over 70: OR = 4.83, CI 3.94–5.91, β = 1.57, se(β) = 0.10, all other age groups – taking 'over 70 years' as a reference – are also significant)

A similar comparison between women and men does not show major differences concerning avoiding or delaying physician visits. Concerning 'per capita income', though, a clear trend emerges (see Figure 3): In autumn 2005, i.e. the survey with the highest percentage of delayers and avoiders, this percentage is 67.9% among those with very little income (less than 600 Euro), compared to 'only' 42.6% among those with very high income (more than 1,300 Euro). This difference is rather stable over time, i.e. these two curves remain rather parallel between spring 2004 and autumn 2006 (OR including all waves, univariate logistic model = 2.68, CI 2.32–3.10, β = 0.99, se(β) = 0.07). Comparing the other income groups with the reference 'more than 1,300 Euro' also reveals significant ORs. The three middle income groups are combined into one curve in this Figure, as the three separate curves more or less overlap. As illustrated by Figure 4, a very similar picture can be seen when the percentages avoiding or delaying physician visits is displayed by the difficulty to pay co-payments: The participants who say that it is difficult to spare the money for the co-payments show a much higher percentage than those who say that paying the co-payments is easy. And again the curves remain rather parallel between spring 2004 and autumn 2006 (OR univariate including all waves = 2.0, CI 1.77–2.25, β = 0.69, se(β) = 0.06).

Concerning the variables assessing health and health awareness, interesting associations can be seen as well (not all data shown here in separate Figures). In spring 2006, for instance, 43.45% of participants with strong health awareness delayed or avoided physician contacts, compared to 69.74% of those with little interest in their own health (OR univariate including all waves, little interest versus strong interest = 1.67, CI 1.40–2.00, β = 0.51, se(β) = 0.09). Those who assess their health status as being 'very good' avoid or delay physician contacts twice as often as those who say that their health is poor (OR univariate including all waves = 2.0, CI 1.74–2.27, β = 0.69, se(β) = 0.07). Figure 5 shows the associations by presence or absence of a chronic disease. Not surprisingly, the shape of the curves resembles those of the general health status (SAH), demonstrating that even a high percentage of patients with a chronic disease avoid or delay physician visits due to the extra fee (OR univariate including all waves, no chronic disease versus chronic disease = 1.90, CI 1.73–2.07, β = 0.64, se(β) = 0.05). Finally, those who pay a maximum of only 1% of their income for co-payments (due to a severe chronic disease) avoid or delay physician contacts to a lesser extent than those who pay the standard of 2% (OR univariate including all waves, 2% versus 1% = 2.4, CI = 2.10–2.77, β = 0.88, se(β) = 0.07).

### Multiple logistic regression analyses

The results of the multiple logistic regression analyses are presented in Table 2. They are based on a backward selection process, and the models comprise complete cases only (N = 7,198). The test for multicollinearity does not reveal significant problems (i.e. the variance inflation factor does not exceed the critical value of 5). The correlation between two variables is still rather high though, i.e. between 'difficulties paying the co-payments' on one hand and 'per capita income' on the other. This is why the variable 'difficulties paying the co-payments' is excluded from the models presented below (but major changes occurring when this variable is included are reported in the text). Various interaction terms were tested, but later withdrawn, because no interaction term was statistically significant in the full model.

**Table 2:** Multivariate logistic models (complete cases only)

	**Model 1** outcome: 'delayed or avoided'	**Model 2** outcome: 'delayed or avoided' *subgroup with chronic disease*	**Model 3** outcome: 'delayed'	**Model 4** outcome: 'avoided'
*N*	*7,198*	*3,542*	*7,198*	*7,198*
	odds ratio (95% CI)			
Age (years) under 30	3.46 (2.72–4.39)	3.35 (2.28–4.94)	1.46 (1.13–1.90)	5.44 (3.69–8.01)
Gender male	0.98 (0.89–1.09)	1.13 (0.97–1.31)	0.90 (0.80–1.00)	1.12 (0.98–1.27)
Per capita Income (Euro)				
< 600	2.31 (1.98–2.70)	2.45 (1.90–3.15)	1.45 (1.22–1.72)	2.20 (1.81–2.68)
600–800	1.73 (1.48–2.01)	1.81 (1.45–2.28)	1.41 (1.19–1.67)	1.59 (1.29–1.96)
800–1,000	1.31 (1.11–1.55)	1.46 (1.14–1.87)	1.24 (1.03–1.49)	1.20 (0.95–1.52)
1,000–1,300	1.39 (1.19–1.63)	1.47 (1.17–1.85)	1.27 (1.06–1.51)	1.29 (1.04–1.61)
HAW^a ^little	1.28 (1.05–1.57)	1.94 (1.38–2.74)	0.68 (0.53–0.87)	2.09 (1.66–2.63)
SAH^b ^very good	1.13 (0.95–1.34)	1.22 (0.93–1.61)	1.17 (0.97–1.41)	1.02 (0.81–1.29)
Chronic disease no	1.13 (0.99–1.28)	-	0.84 (0.73–0.96)	1.53 (1.30–1.80)
Maximum co-payment 1% no	1.77 (1.50–2.09)	1.71 (1.43–2.04)	1.57 (1.31–1.88)	1.59 (1.22–2.08)
survey spring 2006	1.95 (1.65–2.31)	1.88 (1.47–2.42)	2.56 (2.12–3.10)	0.86 (0.69–1.07)
c-value	0.69	0.70	0.62	0.72
H/L^c ^p-value	0.49	0.43	0.45	0.31
Pseudo R^2 ^Nagelkerke	0.14	0.13	0.10	0.15
Pseudo R^2 ^McFadden	0.08	0.08	0.03	0.10

^*a *^Health awareness, ^*b *^Self assessed health, ^*c *^Hosmer Lemeshow test

Comparison groups: age: over 70; gender: female; per capita income: > 1,300 Euro; HAW: strong; SAH: poor; chronic disease: yes; max. co-payment of 1%: yes; survey: spring 2004

Four models are presented here, two models with the outcome variable 'delayed or avoided physician visits' (see models 1 and 2), one model restricted to the outcome 'delayed' (model 3) and one model restricted to the outcome 'avoided' (model 4). Also, one model is restricted to the subgroup of those with a chronic disease (model 2). Due to formal integrity, the variables 'gender' and 'self assessed health' are included in the Table as well, although they do not show statistically significant associations with the dependent variables.

People under 30 years of age are more than three times (OR = 3.46, CI 2.72–4.39, β = 1.24, se(β) = 0.12) more likely to avoid or delay physician visits because of the practice charge than people older than 70 years (model 1). For those aged 31 to 40 years it is 2.47 (CI 1.97–3.11) times, for those aged 41 to 50 years 1.94 (CI 1.56–2.43) times, for those aged 51 to 60 years 1.89 (CI 1.52–2.36) times, and for those aged 61 to 70 years 1.46 (CI 1.18–1.80) times (odds ratios not presented in the Table). Thus, a clear age gradient emerges (as already seen in Figure 2). Very similar odds ratios are derived for age in all other models. The non-significant association with the variable 'gender' can also be seen in all models. Concerning the variable 'per capita income', the odds ratios for all categories are shown in Table 2, since this parameter is the main independent variable in these analyses. Participants with less than € 600 are about 2.3 times more likely to delay or avoid physician visits than those with more than € 1,300 (model 1, OR = 2.31, CI 1.98–2.70, β = 0.84, se(β) = 0.08). This association is even somewhat stronger in the subgroup of the participants with a chronic disease (model 2). If the variable 'difficulties with co-payments' is included, the odds ratios of 'income below € 600' decreases to 1.54 (CI: 1.30–1.83) in model 1 and 1.61 (CI: 1.23–2.12) in model 2. Some of the odds ratios for the other income groups are clearly significant as well and, broadly speaking, an increasing odds ratio with decreasing income can be seen. The odds ratios for 'difficulties paying co-payments' versus 'no difficulties' are 2.67 (CI: 2.29–3.11) in model 1 and 2.62 (CI: 2.11–3.27) in model 2 (not shown in Table 2).

'Health awareness' plays a bigger role in model 2 than in model 1, while 'self assessed health' is not significant in both models. The participants who contribute a maximum of 2% of their annual income to co-payments are 1.77 times (model 1, β = 0.57, se(β) = 0.08) or 1.71 times (model 2, β = 0.54, se(β) = 0.09) more likely to delay or avoid physician visits than those who need to pay only 1% (due to a severe chronic disease). As has been discussed above, the prevalence for delaying or avoiding physician visits changes considerably between the six waves. We tried to adjust for this by including an additional variable 'survey spring 2006 versus survey spring 2004' (i.e. by comparing the first survey with the last available survey that has been conducted in the same time of the year). The results show that the prevalence is about 2 times as high in spring 2006 (OR = 1.95, CI 1.65–2.31, β = 0.67, se(β) = 0.09) compared with spring 2004.

In other models (Tables not shown here), we excluded the variables 'survey' and 'maximum co-payment of 1%', but the odds ratios for the key variable 'per capita income' do not change very much. For the lowest income group, for example, they are 2.24 (CI: 1.92–2.61) including all participants, and 2.25 (CI: 1.76–2.88) including only those with a chronic disease. Also, we conducted additional analyses with all missing values included by separate dummy variables, but again the odds ratios did not change remarkably. For instance, the probability of people with less than € 600 to avoid or delay physician contacts changes from 2.31 (model 1) to 2.32 (CI: 1.99–2.71). It is also important to stress that the dummy variable 'no information on income' is not significantly related to avoiding or delaying physician contacts (OR = 1.24).

The analyses differentiating between the outcome 'delayed' on one hand and 'avoided' on the other yield some additional information. The associations for age, per capita income, health awareness and absence of a chronic disease are especially large for the outcome 'avoided' (i.e. the more extreme outcome).

The c-values of all models are satisfactory. Likewise, all Hosmer Lemeshow tests are not significant, indicating good model appropriateness. Nagelkerke's and McFadden's Pseudo R^2 ^are very small, though, indicating that the selected independent variables are not sufficient for fully explaining the variance of the outcome variable.

## Discussion

The prevalence of 'delayed or avoided physician visits' increased from 35.8% in spring 2004 to 46.8% about six months later, stayed high until spring 2006 and decreased to 35.7% in autumn 2006. Also, most subgroups of the respondents (e.g. defined by age and income) showed a very similar increase and decrease over the course of these six surveys. It can be concluded that it took some months for the reaction to this new regulation to fully develop, that about 2^1^/_2 _years after its implementation its effect started to diminish, and that the social differences in this reaction remained rather stable in this period.

However, it is difficult to fully explain the observed time trend and especially the sharp decline between spring and autumn 2006, because avoiding or delaying physician visits is a very complex issue with many influencing factors. The question concerning the physician charge was identical in all surveys and no major political change took place in this time period. It has to be stressed, though, that the association of primary importance here (e.g. between income on one hand and delayed or avoided physician visits on the other) remains surprisingly stable in all six cross-sectional surveys (with different persons included in each survey). If possible, future studies should be based on a longitudinal design, as this allows for a much more precise assessment of time trends (and causal effects), of course.

Younger people were more likely to avoid or delay a physician visit than older. This is rather plausible, as usually they are healthier than older people and need to see a physician less urgently. The age gradient can still be seen after controlling for self assessed health and the presence of a chronic disease. This is probably due to the fact that the need to see a physician is not fully adjusted for by these two health variables. As expected, those who state that their health is poor or that they have a chronic disease delay or avoid a physician visit less often than those who are healthier (see Figure 5). Controlling for the other variables in the logistic regressions, though, the influence of these health indicators is rather small. It is significant only in the model focussing on the outcome 'avoided' (see Table 2, model 4). A third possibility to assess the presence of a chronic disease is presented by the variable 'maximum co-payment 1%'. Whereas about 49% of all participants state that they have a chronic disease, only about 14% state that they are exempt from the standard of 'maximum co-payment 2%' (see Table 1), clearly pointing to the fact that the indicator 'maximum 1%' indicates the presence of more severe diseases. This is probably the reason why this indicator for health shows the strongest association with the dependent variables.

Concerning the independent variable of primary importance here, i.e. income, the bivariate analyses clearly show that delaying or avoiding physician visits is reported most often in the lowest income group (see Figure 3). The association can also be seen in the multivariate analyses. In the subgroup of respondents having a chronic disease, for example, this reaction is reported in the lowest income group 2.45 times more often than in the highest income group. Also, a dose response association can be seen here (i.e. decreasing odds ratios with increasing income).

The fact that the Pseudo R^2 ^values are rather small indicates that the variables included here are not sufficient to fully explain the variance of the outcome 'delayed/avoided'. We selected the independent factors after testing them for univariate significance and could of course only choose among those characteristics that had been included in the original study questionnaire. It is quite probable that a number of other aspects play a role in a patient's decision to consult a physician or not, namely his or her time budget, good or bad past experiences, travel distances to the physician's office, the physician-patient relationship or language barriers.

The results of the logistic regression can be used to calculate prognostic scores. Taking model 1 (Table 2), for example, the minimal risk score is obtained for a male person, who is more than 70 years old, earns more than 1300 € per month, has strong health awareness but poor self assessed health, has a chronic disease, pays only 1% for co-payments and has participated in the survey of spring 2004. His probability to delay or avoid a physician contact because of the practice charge is only 7.38%, whereas it is 81.62% for a young woman with the maximum risk profile. Thus, the odds ratio of a person at maximum risk (as compared with a person at minimum risk) reaches 55.72. These calculations are just intended to illustrate best and worst case situations. In reality, very few people belong to these extreme categories (in our study 8 old men and 5 young women).

The data used in this analysis are obtained by consecutive cross-sectional surveys. There is no follow up information per person over time, and therefore it is difficult to assess causality. It seems to be rather plausible, though, to assume that income has a causal effect on avoiding or delaying physician contacts due to the practice charge. Another problem is of primary concern here: The need to see a physician could only be assessed in a crude way, i.e. by taking into account measures of health and chronic disease. The final objective should be to analyse the health effects of delaying or avoiding physician visits, and this will only be possible with a better assessment of the need to see a physician, and with follow up information on health. Future studies should try to fill this gap. The present analysis can just point to the fact that negative health consequences of the new 'Praxisgebuehr' are probably most prevalent in the lowest income group (thus increasing health inequalities). Another limitation of the study is the fact that we could not assess if a person was exempt from the physician fee during the time period asked for in the survey (past three months). Most people accumulate their bills and ask for refund by the end of the year, but other may apply for exemption before the end of the year; these details are not asked for in the questionnaire, though.

Co-payments have been introduced in many industrial countries in order to minimize health insurance expenditures, to close budget gaps in the public health sector and to restrict moral hazard. As mentioned above, the concept of insured people 'over-using' insurance services is derived from insurance theory. M.V. Pauly hypothesised in 1968 that such 'excessive' demands could be depleted effectively by monetary hurdles such as co-payments [21]. However, health care systems are very different from other markets regulated by supply and demand. Patients cannot choose treatments and medications like TV sets or beer brands, but have to rely on physician's decisions and recommendations. Moreover, medical services and products are rarely a matter of taste like luxury goods.

In particular, socio-economically disadvantaged people tend be less concerned about their own health [4], even if medical services are offered free of charge (such as preventive medical services in Germany), and co-payments can be an important additional financial hurdle. H. Reiners argues that the moral hazard concept is utterly misplaced in the public health sector, and that it is more plausible to assume that physicians are 'over-using' the system by inducing demand [22]. The moral hazard argument has initiated highly controversial discussions in many countries, since co-payments and cost sharing schemes are frequently applied as a 'one-size-fits-it-all' tool in health politics, often disregarding the potential for jeopardizing health care especially for the poor. It is very difficult, of course, to determine a level of co-payment that discourages unnecessary utilization of services, and that does not discourage patients from seeking medical services they really need [23]. A growing body of literature suggests that co-payments may adversely affect health outcomes [24]. To date, the RAND-Study, conducted in California during the 1970s, is regarded as a fundamental investigation on moral hazard in health insurance [25]. 5,809 US citizens were randomly assigned to 14 different health insurance contracts (with different co-payment modalities) and their consequent behaviour was documented for three to five years. One interesting result was that patients with higher co-payments abstained from necessary physician visits and had worse health outcomes in the end (e.g. concerning their teeth, blood pressure and eye sight). Furthermore, cost sharing had particularly negative effects on people with low income and shortened the lifetime of high risk patients [26]. The negative effect of co-payments especially for socioeconomically disadvantaged groups could also be seen e.g. in studies from Israel [27,28], South Korea [29], France [30,31] and Denmark [32]. In Austria and the Netherlands, some co-payments have been abolished after thorough evaluation, because they turned out to deter socio-economically disadvantaged patients from physician visits [33]. A study in Canada documents that an increase of cost-sharing for prescribed drugs resulted in a decrease in essential medication among poor and elderly patients [34]. A decrease in the utilization of life-sustaining drugs was also found in an international Cochrane review including 21 studies [35]. Thus, there is increasing evidence of serious adverse effects that could ultimately lead to higher health care expenditures.

In a discussion paper on European strategies for tackling social inequities in health, the WHO states that denying access to effective health care is a denial of human rights. Nevertheless, the existence of inequities in access to health care can even be found in the most advanced welfare systems in Europe [36]. A health care system is needed that guarantees basic, affordable health care coverage for all citizens without discriminating specific socio-economic groups. Co-payments may pose a major threat to this principle of solidarity, they could lead to increasing health inequalities, and also to higher health care cost for disadvantaged population groups in the long run. It would be important to assess these potential problems in more detail, and in advance (e.g. by conducting health inequality impact assessments before a reform such as the 'Praxisgebuehr' is implemented). The financial resources are limited, of course, and in some circumstances co-payments could be helpful for adjusting the provision of health care services to health care needs. They should not be an instrument for increasing health inequalities, though.

## Conclusion

In conclusion, the results of our study are in line with other studies, indicating that practice charges for physician visits and other co-payments could jeopardize health care utilization, especially among socially deprived groups. Apparently, even a relatively small amount of money could detain patients from physician visits (and also unsettle the relationship between the patient and the physician).

## Competing interests

The authors declare that they have no competing interests.

## Authors' contributions

JB has kindly provided the data and revised the manuscript. The project was drafted, initiated and supervised by AM, who also contributed to the manuscript.

In the course of her master thesis in Public Health, IMR carried out the statistic analysis, prepared and edited the script. All authors read and approved the final manuscript.

## Pre-publication history

The pre-publication history for this paper can be accessed here:


